# Improving the Thermostability of Lipase ATL by Engineering Disulfide Bonds in Flexible Regions

**DOI:** 10.3390/foods15213866

**Published:** 2026-10-29

**Authors:** Lilang Li, Ruiguo Cui, Dong Huang, Naiyong Xiao, Fengjun Li

**Affiliations:** 1 Guangdong Provincial Key Laboratory of Aquatic Product Processing and Safety, Guangdong Province Engineering Laboratory for Marine Biological Products, Guangdong Provincial Engineering Technology Research Center of Seafood, Guangdong Provincial Engineering Technology Research Center of Prefabricated Seafood Processing and Quality Control, College of Food Science and Technology, Guangdong Ocean University, Zhanjiang 524088, China; langll@gdou.edu.cn (L.L.); h3064866856@163.com (D.H.); nyxiao@gdou.edu.cn (N.X.); 2 Collaborative Innovation Center of Seafood Deep Processing, Dalian Polytechnic University, Dalian 116034, China; 3 College of Food Science and Technology, Hebei Normal University of Science & Technology, Qinhuangdao 066612, China; ruigc_hnust@163.com

**Keywords:** lipase, disulfide bonds, molecular dynamics simulations, functional lipid

## Abstract

Lipases are versatile biocatalysts for the synthesis of functional lipids, but their industrial application is frequently constrained by limited thermostability. In this study, disulfide bond engineering targeting flexible regions identified by molecular dynamics (MD) simulations was employed to enhance the thermostability of lipase ATL from *Aspergillus tamarii*. The combined mutant Mut2D harboring the disulfide bonds A46C-N68C and L263C-G271C exhibited a *T_m_* increase of 5.83 °C and a 23.9-fold improvement in half-life at 45 °C compared to the wild-type ATL. The catalytic efficiency (*k_cat_*/*K_m_*) of Mut2D for pNP-butyrate, pNP-caprate and pNP-stearate increased by 34.7%, 27.0% and 39.0%, respectively, compared to the wild-type. Comparative MD simulations showed that the introduction of the two disulfide bonds increased the overall conformational rigidity of the enzyme, which likely accounts for the increased thermostability. In diacylglycerol (DAG) synthesis, Mut2D achieved a 4.8% higher DAG content with a faster reaction rate compared to the wild-type at their respective optimum temperatures. This study paves the way for the industrial application of ATL and provides valuable insights for enhancing the thermostability of other lipases.

## 1. Introduction

Functional lipids have attracted increasing attention in food and health-related applications, because their molecular composition and structure can be tailored to improve physicochemical and biological properties, with diacylglycerols (DAGs), omega-3-rich lipids, and human milk fat substitutes, among others, being prominent product classes [1]. Lipases are key versatile biocatalysts capable of catalyzing hydrolysis, esterification, transesterification, and acidolysis with high substrate, regio-, and stereoselectivity, enabling targeted lipid restructuring as opposed to non-specific modification [2]. Consequently, lipase-catalyzed processes have become a pivotal approach for the synthesis of functional lipids. Compared with conventional chemical processing, lipase-catalyzed reactions display various advantages, including mild reaction conditions, environmental friendliness, and high product purity [3].

Thermostability is a critical parameter for evaluating enzyme performance in industrial applications, including preparing functional lipids [4]. Enzymes with high thermostability display enhanced kinetic and operational stability under increased reaction temperatures [5]. The advantageous enzyme property not only accelerates reaction rates, but also reduces the risk of microbial contamination and facilitates the effective recycling of the biocatalyst, consequently reducing overall manufacturing costs [6]. However, naturally occurring enzymes frequently suffer from inadequate thermostability, which severely restricts their practical utility in industrial applications.

Over the last decade, computational approaches have gained substantial traction as efficient tools for engineering enzyme thermostability, providing significant benefits over traditional directed evolution protocols that depend upon large-scale mutagenesis libraries and high-throughput assays. Unlike such time-consuming and costly procedures, rational design, driven by structural and computational predictions, allows swift identification of favorable mutations with reduced experimental workload [7]. The computational design approaches have greatly accelerated the identification of mutations that improve enzyme thermostability. Recently, molecular dynamics (MD) simulations have been increasingly applied to determine conformationally flexible regions within protein structures [8]. Notably, the conformational dynamics of such unstable regions can be restrained via the introduction of additional stabilizing interactions, including hydrogen bonds, π-π interactions, salt bridges, and disulfide bonds, representing a highly effective strategy for enhancing enzyme thermostability [9]. Among these interactions, disulfide bonds, which are covalent linkages generated from the thiol groups of two cysteine residues. They reduce the conformational entropy of the unfolded state, thereby counteracting reversible unfolding, and they slow the unfolding rate, consequently hindering denaturation [10]. The design of disulfide bonds has been applied to improve the thermostability of various enzymes, including phospholipase [9], alkaline κ-carrageenase [11], lipase [12], D-psicose 3-epimerase [13] and zearalenone hydrolase [14].

The lipase from *Aspergillus tamarii* (ATL) exhibits high regioselectivity, whereas its limited thermostability hampers its industrial application [15,16]. In this study, the engineering disulfide bond strategy was employed to improve the thermostability of lipase ATL. Initially, MD simulation was utilized to identify the conformationally flexible regions of lipase ATL, which were subsequently targeted for disulfide bond engineering. Single beneficial disulfide bonds were identified through biochemical assays, and subsequently combined to generate mutants with further enhanced thermostability. Furthermore, homology modeling coupled with MD simulation was employed to elucidate the molecular basis underlying the improved thermostability at the atomic level. Finally, the catalytic effect of the mutant in DAG preparation was investigated and compared with wild-type ATL. Our study not only provides valuable insights for thermostability enhancement of other members within the lipase superfamily, but also facilitates the broader application of ATL in food and pharmaceutical industries.

## 2. Materials and Methods

### 2.1. Materials, Reagents, Plasmids and Strains

The gene encoding the wild-type lipase ATL (GenBank accession no. EU131679), fused with an N-terminal 6×His-tag, was codon-optimized for *P. pastoris* and chemically synthesized by Junji Biology (Suzhou, China). The synthetic fragment was inserted into the pPICZαA expression vector through EcoRI and SalI restriction sites, generating the recombinant plasmid pPICZαA-ATL. *E*. *coli* DH5α (Tsingke Biotechnology, Beijing, China) was employed for the site-directed mutagenesis, whereas *P. pastoris* X33 from Invitrogen (Carlsbad, CA, USA) was utilized as the expression host.

### 2.2. Homology Modeling and Molecular Dynamics Simulation

To delineate the flexible regions susceptible to thermal unfolding, the MD simulations were executed using GROMACS 2024.5 under the AMBER ff14SB force field [17]. The crystal structure of lipase ATL was obtained from RCSB (PDB ID: 5XK2). The crystal structure of lipase ATL was solvated in a dodecahedral box filled with TIP3P water, while a minimum distance of 1.2 nm was preserved between the protein surface and the box boundary. An appropriate number of sodium counterions was added to neutralize the system. After the energy minimization process, the system was equilibrated sequentially in the NVT ensemble for 100 ps at 473 K and in the NPT ensemble for 100 ps at 1 bar. A production MD run of 100 ns was then conducted with an integration time step of 2 fs at 473 K and 1 bar. The root-mean-square fluctuation (RMSF) of the Cα atoms was computed with GROMACS built-in tools to identify residues exhibiting high conformational flexibility.

### 2.3. Computational Design of Disulfide Bonds

Candidate residue pairs for de novo disulfide bond introduction were predicted using Disulfide by Design 2 (DbD2), SSBOND and BridgeD based on the structure of ATL. In DbD2 calculations, the χ3 torsion angle was constrained to −87° ± 45° or +97° ± 45°, while the Cα–Cβ–Sγ angle was maintained at 114.6° within a ±10° tolerance. SSBOND and BridgeD were used with the default settings [18]. Predicted pairs residing within the high-RMSF flexible regions identified by MD simulation were prioritized.

### 2.4. Site-Directed Mutagenesis

Cysteine substitutions were introduced using the PrimeSTAR^®^ Max DNA Polymerase (TaKaRa, Dalian, China). The recombinant plasmid pPICZαA-ATL was used as the template, and mutagenic primers were designed with the NEBaseChanger online tool. All primer sequences are listed in Table S1. Amino acid residues are numbered according to their positions in the amino acid sequence, rather than by PDB numbering. The PCR products were treated with the DpnI (TaKaRa, Dalian, China) enzyme for 1 h at room temperature to degrade the methylated parental template. The resulting mixtures were transformed into chemically competent *E. coli* DH5α. Positive clones were recovered on low-salt LB agar plates supplemented with 25 μg·mL^−1^ Zeocin, and the presence of the desired mutagenesis was verified via Sanger sequencing (Tsingke Biotechnology, Beijing, China).

### 2.5. Expression of ATL and Its Mutants in P. pastoris X33

The sequence-verified wild-type and mutant plasmids were linearized with MssI (Thermo Fisher Scientific, Waltham, MA, USA) and purified with an Omega CyclePure kit (Norcross, GA, USA). Approximately 5–10 μg of linearized DNA was electroporated into competent *P. pastoris* X33 cells. Transformants were selected on YPD agar plates (1% yeast extract, 2% peptone, 2% glucose, 2% agar) supplemented with 100 μg·mL^−1^ Zeocin after incubation at 30 °C for 3–4 days.

Individual colonies were transferred into 10 mL of YPD medium supplemented with 100 μg·mL^−1^ Zeocin and cultivated at 30 °C with orbital shaking at 220 rpm until the OD_600_ reached 2.0–3.0. Cells were harvested by centrifugation at 3000× *g* for 5 min and resuspended in 50 mL of buffered methanol-complex (BMMY) medium (100 mM potassium phosphate, pH 6.0, 1% yeast extract, 2% peptone, 1.34% yeast nitrogen base) in 250 mL baffled flasks to induce protein expression. To maintain induction, methanol was supplemented at 24 h intervals to reach a final concentration of 0.5%. The culture was incubated at 28 °C and 220 rpm for 72 h, after which the supernatant was collected by centrifugation at 8000× *g* for 10 min at 4 °C.

### 2.6. Purification of Recombinant Proteins

The clarified supernatant was loaded onto a Ni-NTA Sepharose column (GE Healthcare, Uppsala, Sweden). After washing with 20 mM sodium phosphate buffer (pH 7.0) containing 300 mM NaCl and 50 mM imidazole, the His-tagged enzyme was eluted with the same buffer but containing 250 mM imidazole. The eluate was immediately desalted on a HiPrep 26/10 column (GE Healthcare) pre-equilibrated using 20 mM sodium phosphate (pH 7.0). Protein integrity and purity were monitored by 12% SDS-PAGE.

### 2.7. Thermal Stability Screening by DSF

The thermal stability of wild-type ATL and its cysteine mutants was evaluated by differential scanning fluorimetry (DSF) using SYPRO Orange (Sigma-Aldrich, Saint Louis, MO, USA) as an extrinsic fluorescent probe [19]. In a total volume of 25 μL, 20 μL of purified enzyme (0.25 mg·mL^−1^) and 5 μL of 50-fold SYPRO Orange dye were mixed. Fluorescence intensity was monitored on a Bio-Rad CFX 96 real-time PCR system (Hercules, CA, USA) across a linear temperature gradient from 20 to 95 °C at a heating rate of 1 °C·min ^−1^. FRET was employed as the detection channel in the detection procedure. The melting temperature (*T_m_*) was derived by fitting the first-derivative fluorescence curve to a Boltzmann sigmoidal model using GraphPad Prism 9.5 software. Additionally, protein quantification of the purified enzymes was performed employing a BCA assay kit (Sango Biotech, Shanghai, China) in accordance with the manufacturer’s instructions.

### 2.8. Enzymatic Characteristics

The purified lipase activity was determined using the p-nitrophenyl-caprate (pNP-caprate) method [20]. The standard assay mixture contained 80 μL of sodium phosphate buffer (20 mM, pH 6.0), 10 μL of 10 mM pNP-caprate, and 10 μL of purified lipase. The mixture was incubated at the specified temperature for 10 min and was stopped by the addition of 100 μL of 1% (*w*/*v*) SDS. Then the absorbance of the reaction termination solution was recorded at 405 nm. One unit of lipase activity corresponds to the quantity of enzyme required to liberate 1 μmol of p-nitrophenol per minute under the assay condition. The calibration curve used to quantify the released p-nitrophenol under the condition was determined, and then the molar extinction coefficient of p-nitrophenol was calculated (1272.1 M^−1^ cm^−1^).

For thermostability assessment, purified enzymes (0.1 mg·mL^−1^) in 20 mM sodium phosphate buffer (pH 6.0) were incubated at 45 °C, and aliquots were withdrawn at predetermined intervals to measure residual activity at 40 °C. The *t*_1/2_ was obtained by fitting the residual activity versus incubation time to a first-order decay model. The optimal temperature was evaluated by measuring activity at temperatures ranging from 25 to 60 °C. The optimal pH was evaluated by measuring activity at different pH values from 2.0 to 9.0 at 40 °C. For the assay to evaluate pH stability, the lipase samples were incubated in different pH buffers at 25 °C for 24 h, and then the enzyme activity was determined at 40 °C. The following buffers (20 mM) were used: sodium citrate buffer (pH 3.0–5.0), sodium phosphate buffer (pH 6.0–7.0), Tris-HCl buffer (pH 8.0–9.0), and sodium carbonate-bicarbonate buffer (pH 10.0).

The kinetic parameters, including the catalytic constant (*k_cat_*), Michaelis constant (*K_m_*), and specificity constant (*k_cat_/K_m_*), were evaluated using varying concentrations of pNP-butyrate, pNP-caprate and pNP-stearate at 45 °C and pH 6.0 for 10 min. The parameters were calculated via non-linear regression analysis in GraphPad Prism 9.5.

### 2.9. The Verification of Disulfide Bond Formation

The successful formation of engineered disulfide bonds was confirmed by quantifying free sulfhydryl groups using Ellman’s assay [21]. Purified enzyme solutions (0.3 mg/mL, 0.5 mL) prepared in 50 mM Tris-HCl supplemented with 5 M guanidine hydrochloride were combined with 2 mL of 0.1 mM 5,5′-Dithio-bis-(2-nitrobenzoic acid) DTNB reagent. Following a 20 min incubation at 37 °C, absorbance was recorded at 412 nm, and thiol concentrations were calculated using the extinction coefficient of 13,600 M^−1^ cm^−1^.

### 2.10. The Synthesis of DAG

The synthesis of DAG was carried out according to the previous method with minor modification [22]. The reaction system, comprising glycerol and oleic acid at a molar ratio of 7:1, water (5%, *w*/*w*, relative to the total substrate mass), and the purified enzyme, was added to a 25 mL flask. The resulting reaction mixture was incubated at different temperatures in an air-bath shaker at 200 rpm. Aliquots were withdrawn at predetermined time intervals. Product composition was analyzed by high-performance liquid chromatography according to the description of Wang et al. [22]. The chromatographic system (Waters 2695, Milford, CT, USA) was fitted with a refractive index (RI) detector (Waters 2414, Milford, USA) coupled with a Phenomenex Luna analytical column (4.6 mm × 250 mm, 5 µm, Torrance, CA, USA). Triglyceride (TAG), oleic acid, 1,3-DAG, 1,2(2,3)-DAG, 1(3)-monoglyceride (MAG) and 2-MAG showed peaks at 3.0, 4.3, 5.0, 6.3, 26.1, and 33.1 min, respectively. The calibration curves of the substances were constructed, and the quantitative analysis was conducted according to the curves as described previously [23]. DAG content was expressed as the mass percentage of DAG relative to the total mass of all lipid species (TAG, DAG, MAG, and oleic acid) in the esterification products [23].

### 2.11. Statistical Analysis

All experimental assays were performed in three independent replicates, and the obtained data are expressed as mean ± standard deviation. Statistical comparisons were carried out using SPSS 26.0.

## 3. Results and Discussion

### 3.1. Flexible Region Identification and Disulfide Bond Design

**Figure 1:**
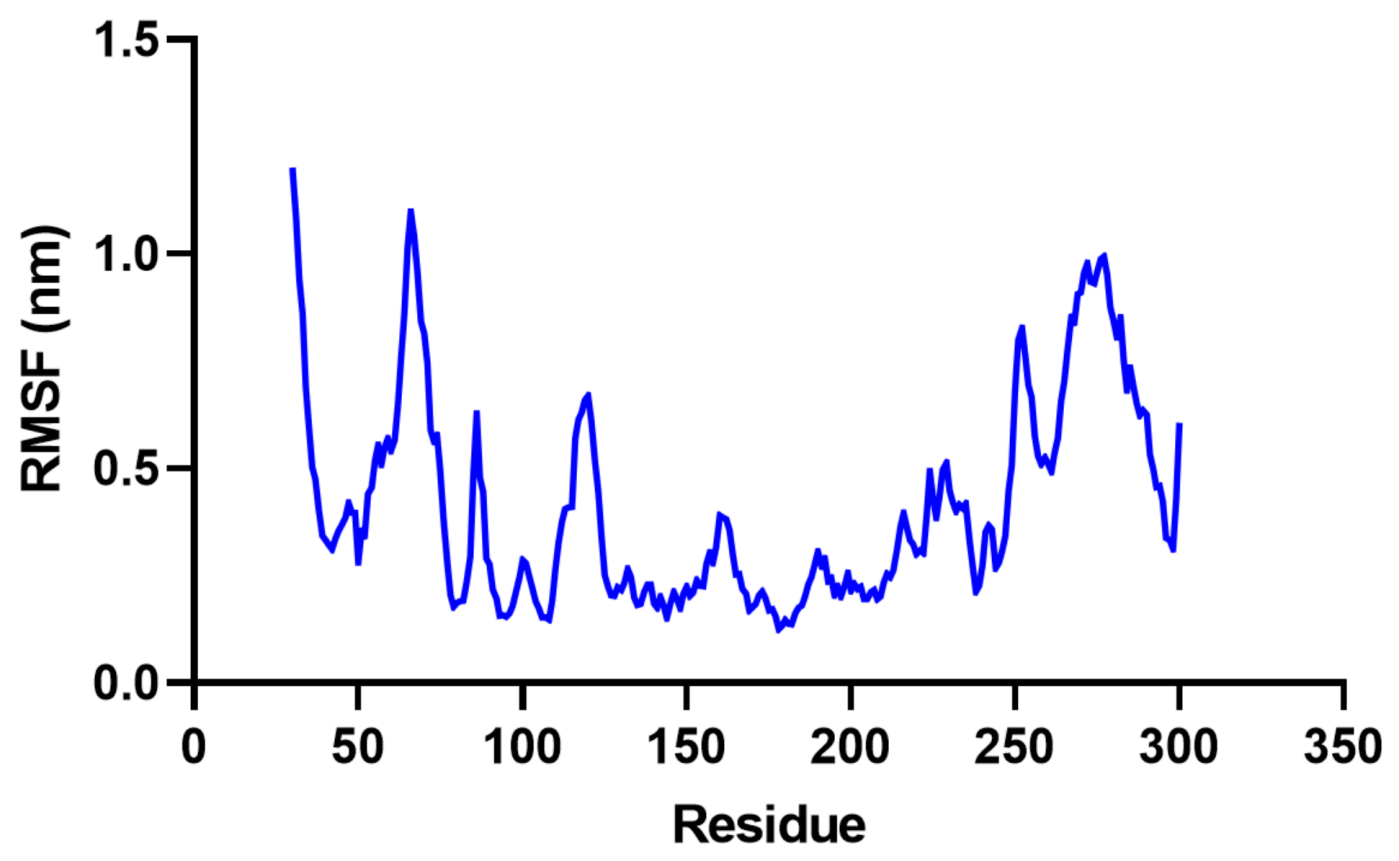
The RMSF calculation of Cα atoms for lipase ATL at 473 K.

The MD simulation was conducted at a hyperthermic temperature of 473 K to accelerate the unfolding process of lipase ATL. The RMSF of Cα atoms was calculated to quantify the positional mobility of each residue along the simulation trajectory to determine the conformationally flexible regions of lipase ATL. Higher RMSF values indicate greater local flexibility [24]. As shown in Figure 1, multiple residues exhibited peak RMSF values exceeding 0.6 nm, including the N-terminal residue as well as residues 25, 65, 86, 120, 252, and 277. Therefore, the regions corresponding to these residues were considered as key positions for the disulfide bond introduction.

The computational tools DbD2, SSBOND and BridgeD were employed to predict residue pairs capable of forming disulfide bonds. The formation of disulfide bonds requires that the residue pairs satisfy specific geometric criteria; thus, not all of these critical regions are suitable for disulfide bond introduction, and the disulfide bonds could be introduced into the residues 50–90 region and residues 250–300 region. Ten candidate disulfide bond mutants were selected for experimental validation, including mutants A46C-N68C, A56C-S82C, V66C-A297C, S84C-A92C, T89C-A143C, A249C-Q259C, D251C-W290C, L263C-G271C, G265C-N268C and F269C-G274C. All disulfide bonds were introduced into the lipase ATL via PCR-based mutagenesis, and the sequences of the mutants were verified by Sanger sequencing.

### 3.2. Expression and Purification of Wild-Type ATL and Disulfide Bond Mutants

Recombinant plasmids harboring wild-type ATL and all mutants were introduced into the expression host for heterologous protein expression. Following induction, all enzyme samples were harvested and purified via Ni-NTA affinity chromatography, utilizing the N-terminal 6×His-tag fused to the target proteins. SDS–PAGE analysis showed a single distinct band, consistent with the predicted molecular weight of ATL for each sample, without any detectable contaminant bands (Figure S1). The high purity of all prepared enzyme samples ensured reliable biophysical characterization in subsequent assays.

### 3.3. Identification of Stabilizing Disulfide Bonds

The thermodynamic stability of wild-type ATL and each disulfide bond mutant was evaluated by measuring the melting temperature (*T*_m_) using the DSF method with SYPRO Orange as the hydrophobic fluorescent probe. *T*_m_ was defined as the temperature at which 50% of the protein undergoes unfolding [25]. The *T*_m_ values and corresponding changes relative to wild-type ATL (Δ*T*_m_) are summarized in Table 1. Among the ten disulfide bond mutants, four displayed a stabilizing effect with Δ*T*_m_ > 1.0 °C. Wild-type ATL exhibited a *T*_m_ value of 50.68 °C, whereas mutants S84C-A92C, A249C-Q259C and L263C-G271C showed *T*_m_ values of 52.19, 52.99, and 52.84 °C, respectively, corresponding to increases of 1.51, 2.31, and 2.16 °C (*p* < 0.05). The most pronounced stabilization was observed for mutant A46C-N68C, with a *T*_m_ value of 54.77 °C, which was 4.09 °C higher than that of wild-type ATL (*p* < 0.05).

The kinetic stability of the mutants was further assessed by measuring the half-life (*t*_1/2_) at 45 °C. Wild-type ATL displayed a *t*_1/2_ value of 28.7 min (Table 1). In comparison, the mutants S84C-A92C, A249C-Q259C and L263C-G271C showed *t*_1/2_ values of 130.2, 242.6, and 197.3 min, which were approximately 4.5-, 8.5- and 6.9-fold (*p* < 0.05) longer than that of the wild-type, respectively. The most stabilizing mutant, A46C-N68C, extended the *t*_1/2_ value to 311.5 min, which increased to 10.9-fold compared with wild-type ATL (*p* < 0.05). These results indicate that both thermodynamic and kinetic stability of mutants A46C-N68C, S84C-A92C, A249C-Q259C and L263C-G271C were enhanced.

The optimal temperature (*T*_opt_) was further assessed for the beneficial variants (Table 1). All mutants S84C-A92C, A249C-Q259C, and L263C-G271C retained a *T*_opt_ of 45 °C, identical to that of the wild-type ATL, while the *T*_opt_ value of A46C-N68C was increased to 50 °C. This suggests that the enhanced structural rigidity conferred by the introduction of the disulfide bond A46C-N68C necessitates higher thermal energy to achieve maximal catalytic efficiency of the enzyme.

**Figure 2:**
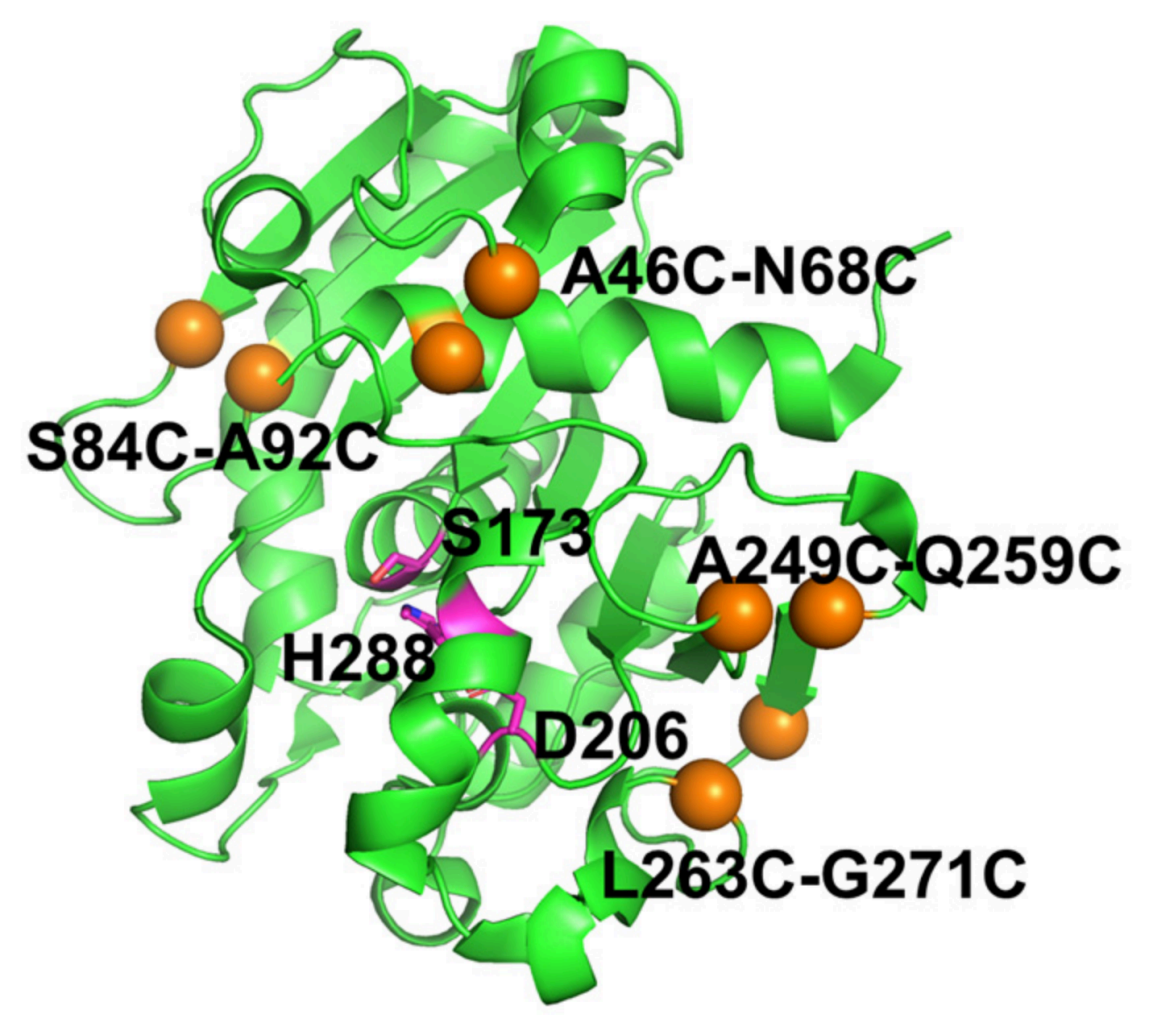
Locations of stabilizing disulfide bonds. The substituted residues are shown as orange spheres, while the catalytic residues S173, D206 and H288 are shown as magenta sticks.

To confirm that the observed stability changes originated from disulfide bond formation rather than the amino acid substitution itself, free thiol content was quantified for wild-type ATL and the four stabilizing mutants using the Ellman’s assay. The free thiol per molecule of wild-type ATL, A46C-N68C, S84C-A92C, A249C-Q259C and L263C-G271C were 0.023, 0.016, 0.039, 0.028 and 0.027 mol/mol, respectively (Table 2). For each cysteine residue in a protein that does not form a disulfide bond, the theoretical free thiol content/molecule increases by 1 mol/mol. Each mutant contains two additional cysteine residues. If the additional cysteines remain in the reduced state, the theoretical free thiol/molecule would be 2 mol/mol higher than that of the wild-type. Conversely, if all cysteine residues are involved in disulfide bonds, the free thiol/molecule would theoretically be 0 mol/mol greater than that of the wild-type. The free thiol per molecule values of the stabilizing mutants suggest that the extra cysteine residues are successfully oxidized to form the intended disulfide linkages, which account for the improved thermostability of the enzyme. Despite some limitations of the Ellman’s assay, including that some cysteine residues may be inaccessible to the reagent, and that proteins may aggregate following guanidine hydrochloride denaturation, thereby decreasing cysteine accessibility [26], the assay is still commonly used to evaluate disulfide bond formation in mutants because of its rapidity and simplicity [27,28,29]. Disulfide bond A46C-N68C connected the termini of two β-sheets, while disulfide bonds S84C-A92C, A249C-Q259C and L263C-G271C contained one or two cysteine residues positioned in loops (Figure 2).

Disulfide bonds can confer structural stability on enzymes, since breaking these covalent linkages is thermodynamically unfavorable [30]. Furthermore, the cross-links formed by disulfide bonds restrict the mobility of the relevant regions and thus reduce the conformational entropy of the unfolded state [10]. However, introducing extra disulfide bonds into an enzyme has not generally contributed to improving thermal stability [10]. In the present work, three mutants (D251C-W290C, G265C-N268C and F269C-G274C) caused a reduction in *T_m_* by 2.94 to 4.15 °C (*p* < 0.05). This destabilization might be ascribed to the breakage of pre-existing favorable interactions, the formation of unfavorable contacts between amino acid residues surrounding the disulfide bonds, the creation of internal cavities, as well as the disruption of salt bridges and the aberrant solvent exposure of hydrophobic residues [31]. Consequently, when attempting to improve the thermostability of enzymes, the comprehensive impacts of engineering disulfide bonds should be carefully considered.

### 3.4. The Combination of Stabilizing Disulfide Bonds

To further improve the thermostability of lipase ATL, effective disulfide bonds were combined. Considering that the engineering of multiple disulfide bonds severely reduces enzyme expression [32], and given the low expression level of the mutant A249C-Q259C, the disulfide bonds A46C-N68C and L263C-G271C were combined to generate the double mutant A46C-N68C/L263C-G271C (Mut2D). The formation of the two engineered disulfide bonds in Mut2D was verified by Ellman’s assay, which showed that the free thiol content was 0.033 mol/mol (Table 2), indicating that disulfide cross-links were successfully formed.

As shown in Table 1, Mut2D exhibited a *T_m_* of 56.51 °C, which was 1.74, 3.67 and 5.83 °C higher than that of A46C-N68C, L263C-G271C and wild-type ATL, respectively. Consistent with the enhanced *T_m_* value, the *t*_1/2_ value of Mut2D was also markedly improved. At 45 °C, the *t*_1/2_ value of Mut2D reached 685.3 min, which was 2.2, 3.5 and 23.9-fold longer than that of A46C-N68C, L263C-G271C and wild-type ATL, respectively. It suggests that the combination of disulfide bonds A46C-N68C and L263C-G271C shows a synergistic effect on the thermostability of the enzyme. Additionally, the *T_opt_* of Mut2D was investigated. Mut2D showed a *T_opt_* value of 50 °C, which was similar to that of mutant A46C-N68C. This indicates the combination of disulfide bonds A46C-N68C and L263C-G271C has little effect on the *T_opt_* value.

**Figure 3:**
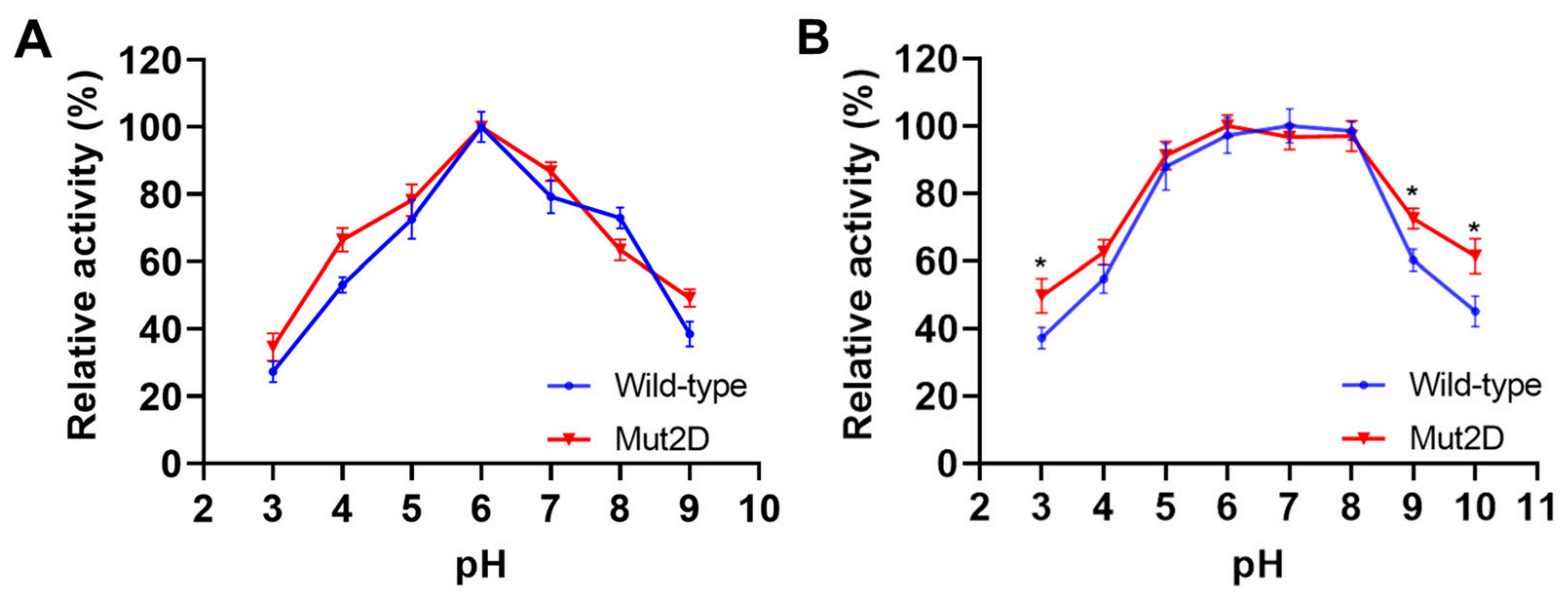
The determination of pHopt (**A**) and pH stability (**B**) for wild-type ATL and mutant Mut2D. Statistical analyses of pH stability between wild-type ATL and mutant Mut2D are carried out with Student’s *t*-test. * indicates *p* < 0.05.

The pH effect on the activity and stability of wild-type ATL and mutant Mut2D was investigated. As shown in Figure 3A, both wild-type ATL and mutant Mut2D exhibited an optimal pH of 6.0, suggesting that the introduction of the two additional disulfide bonds did not affect the optimal reaction pH. Notably, in the pH stability assays, the residual activity of Mut2D at pH 3, pH 9 and pH 10 was 12.4%, 12.2% and 16.3% (*p* < 0.05) higher than that of the wild-type, respectively (Figure 3B). The results indicated that the extra disulfide bonds enhanced the pH stability of the enzyme. Our results are consistent with the view that improved resistance to one type of denaturing factor often correlates with increased tolerance to others [33].

The kinetic parameters of wild-type ATL and mutant Mut2D are shown in Table 3. The *K*_m_ value of Mut2D for pNP-stearate was 12.7% lower than that of the wild-type (*p* < 0.05), and *k_cat_* values of Mut2D for pNP-butyrate, pNP-caprate and pNP-stearate were 22.5%, 16.1% and 21.3% higher than those of the wild-type (*p* < 0.05), respectively. Consequently, *K_cat_ /K_m_* of Mut2D for pNP-butyrate, pNP-caprate and pNP-stearate increased by 34.7%, 27.0% and 39.0%, respectively, compared to the wild-type. This indicates that the catalytic efficiency of Mut2D is higher than that of wild-type ATL. Protein engineering aimed at enhancing thermostability frequently incurs an activity–stability trade-off [34]. However, our results demonstrated that both thermostability and catalytic activity of lipase ATL were simultaneously improved. The results indicate that improving enzyme stability does not invariably conflict with enhancing catalytic activity. The enhancement of both thermostability and catalytic performance could render Mut2D a more attractive catalyst for industrial processes.

### 3.5. MD Simulation Analysis of Mut2D

To elucidate the structural insight underlying the enhanced thermostability of mutant Mut2D at the atomic level, MD simulations were performed for both wild-type ATL and mutant Mut2D at 473 K for 100 ns. The structure of mutant Mut2D was constructed using Modeller 10.8 software.

**Figure 4:**
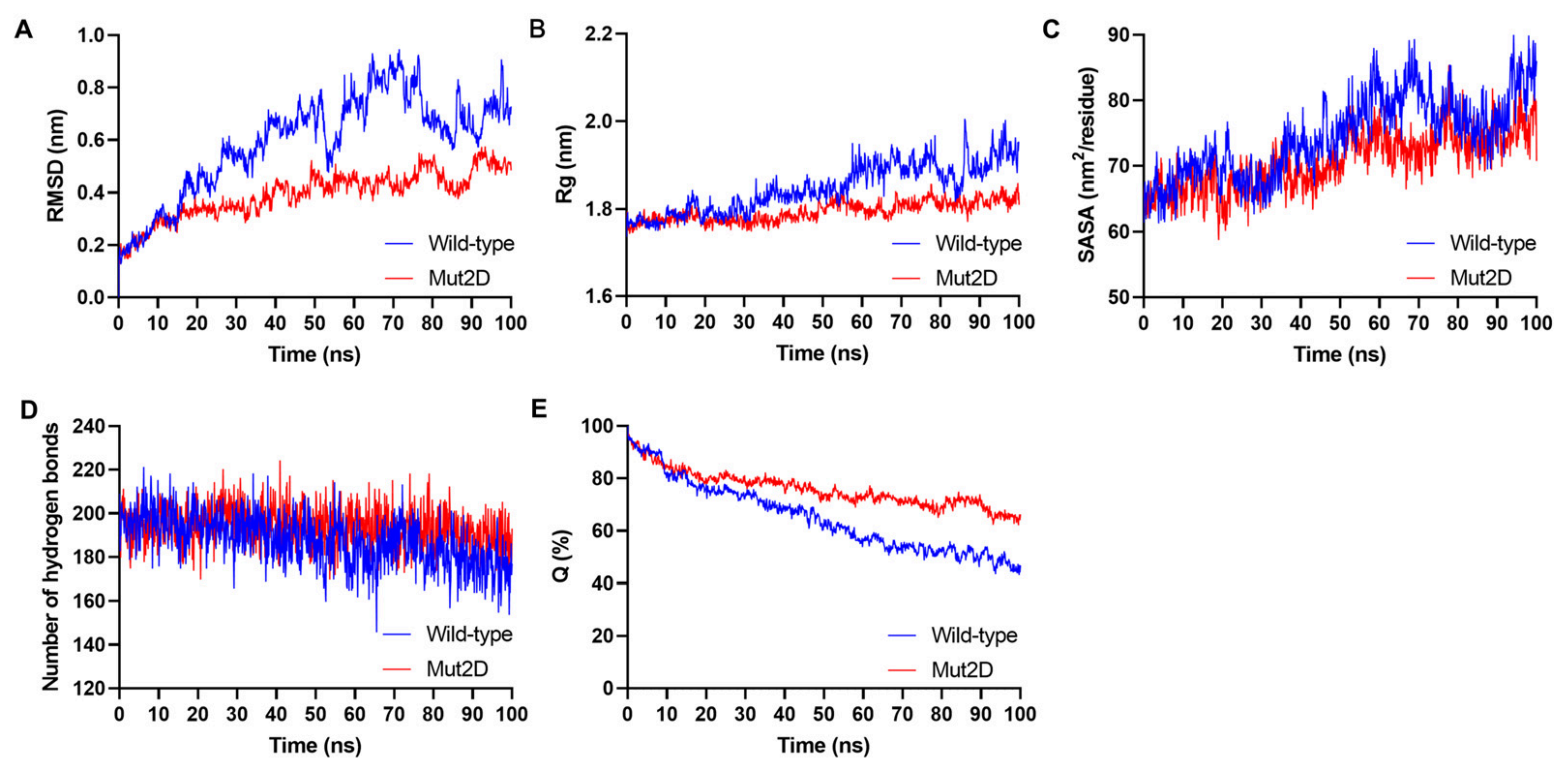
Comparison of MD simulations between wild-type ATL and mutant Mut2D at 473 K. (**A**) RMSD of Cα (**B**) Rg (**C**) hydrophobic SASA (**D**) hydrogen bond number (**E**) Q.

The Cα root-mean-square deviation (RMSD), which reflects the overall deviation of the protein backbone from its initial conformation [35], was monitored throughout the simulation trajectory (Figure 4A). Wild-type ATL exhibited a steady and substantial increase in RMSD, reaching values of approximately 0.5–0.9 nm by the last 40 ns of the simulation. The great fluctuation indicates that a significant structural rearrangement occurred in wild-type ATL. In contrast, mutant Mut2D maintained considerably lower RMSD values, remaining below 0.6 nm throughout the entire simulation. This demonstrates that the combined disulfide bonds substantially constrain the global conformational flexibility of the enzyme.

The radius of gyration (Rg) provides a measure of protein compactness, and a lower and more stable Rg indicates a more tightly packed tertiary structure [36]. As shown in Figure 4B, wild-type ATL displayed a gradual increase in Rg, with the maximum Rg value reaching approximately 2.0 nm, signaling progressive expansion of the protein. However, mutant Mut2D maintained a remarkably stable Rg, fluctuating between 1.78 and 1.82 nm throughout the entire 100 ns trajectory. This result indicates that the disulfide cross-links in Mut2D prevent the thermal expansion of the protein scaffold and preserve a compact fold.

The hydrophobic solvent-accessible surface area (SASA) was calculated to assess the degree of hydrophobic exposure. As depicted in Figure 4C, WT ATL exhibited a pronounced increase in SASA, rising to approximately 86 nm^2^/residue by the end of the simulation, consistent with progressive unfolding and exposure of buried hydrophobic residues. In contrast, the hydrophobic SASA of Mut2D remained at around 78 nm^2^/residue. The lower SASA of Mut2D suggests that the engineered disulfide bonds shield the hydrophobic interior from solvent, thereby reducing the driving force for aggregation and irreversible denaturation.

Hydrogen bonds play a critical role in maintaining protein structures [37]; thus, numbers of intramolecular hydrogen bonds of wild-type ATL and mutant Mut2D were calculated (Figure 4D). At the end of the simulation, the hydrogen bond number of wild-type ATL dropped to about 170, whereas that of mutant Mut2D remained at about 190. The preservation of hydrogen bonding in mutant Mut2D suggests that the additional disulfide bonds can stabilize the hydrogen bond network of the enzyme.

The native contact fraction (Q), which quantifies the proportion of native atomic contacts retained during the simulation [38], was monitored. As shown in Figure 4E, wild-type ATL underwent a steady decline in Q over the 100 ns trajectory, dropping to 44.8% by the end of the simulation. However, mutant Mut2D retained a markedly higher Q value of 66.3% at 100 ns, with a considerably slower decay rate than that of wild-type ATL during the simulation process. This indicates that the dual disulfide bonds preserve a far greater proportion of native inter-residue contacts under thermal stress, effectively retarding the transition from the folded ensemble to the unfolded state.

Protein denaturation generally occurs via a two-stage process. The initial phase involves the reversible expansion of peripheral chains, followed by the exposure of the hydrophobic core and reactive groups, which ultimately leads to irreversible intra- and intermolecular aggregation [39]. In the present work, the introduction of disulfide bonds A46C-N68C and L263C-G271C enhanced the structural rigidity of lipase ATL, which might restrict the expansion of peripheral chains and contribute to preserving critical intramolecular interactions, such as hydrogen bonds, thereby delaying the exposure of hydrophobic patches during unfolding. Consequently, irreversible inactivation was reduced, resulting in greatly improved thermostability of lipase ATL.

### 3.6. Preparation of DAG

DAG is a type of functional lipid that exerts beneficial health effects and unique physiological functions in the human body [40]. In addition, DAGs with both hydrophilic and hydrophobic components have a low hydrophilic–lipophilic balance value, enabling their partitioning in both aqueous and lipid phases, and are widely employed as an emulsifier in food applications [41]. Therefore, it holds considerable interest in the food and biomedical industries. To assess the practical application potential of mutant Mut2D, it was utilized for DAG synthesis.

**Figure 5:**
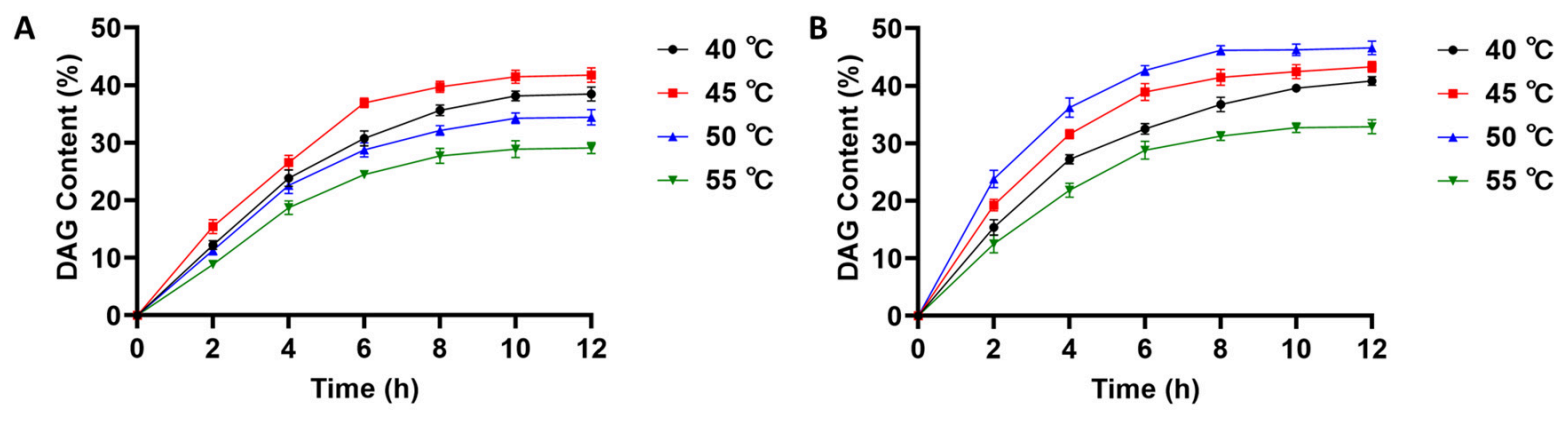
Effect of reaction temperatures on the synthesis of DAG with wild-type ATL (**A**) and mutant Mut2D (**B**).

The enzymatic production of DAG was examined across a gradient of reaction temperatures. As shown in Figure 5, the optimum esterification temperature of wild-type ATL was 45 °C, and the corresponding DAG content was 41.8% after the reaction reached equilibrium (10 h). By contrast, the optimum temperature for mutant Mut2D was elevated to 50 °C, at which the DAG content reached 46.6% after the reaction reached equilibrium (8 h). The DAG content of mutant Mut2D was 4.8% higher than that of wild-type ATL at their respective optimum temperatures (*p* < 0.05) (Figure S2). Notably, at 45 °C, the DAG contents achieved by wild-type ATL were 15.4% and 26.6% at 2 and 4 h, respectively, whereas Mut2D afforded 19.3% and 31.6%, respectively. These results indicate that Mut2D catalyzed DAG formation at a higher rate than wild-type ATL at 45 °C. Lipase ATL exhibited poor thermal stability at 45 °C (Table 1); thus, it might undergo gradual inactivation throughout the reaction process. Mutant Mut2D showed a faster reaction rate than the wild-type at 45 °C, which might be ascribed to its enhanced stability under the reaction conditions. Furthermore, the reaction rate of Mut2D was further elevated by 5 °C. Furthermore, the average DAG productivity of wild-type and Mut2D was calculated at their respective optimum temperatures. At all time points, Mut2D exhibited significantly higher average DAG productivity than wild-type (*p* < 0.05) (Figure S3). Elevated temperatures favor substrate solubilization and reduce the viscosity of the reaction medium [5], thereby accelerating the reaction rate. The higher reaction rate could reduce the time of DAG synthesis. These findings indicate that Mut2D is a promising candidate for subsequent development as an immobilized biocatalyst for the preparation of functional lipids in the industrial process.

## 4. Conclusions

Disulfide bonds were engineered into the flexible regions of lipase ATL to improve its thermostability. The combined mutant Mut2D, harboring two extra disulfide bonds, exhibited simultaneous improvements in both thermostability and catalytic performance. The introduced cross-links increased the global conformational rigidity of the enzyme. Moreover, in the preparation of DAG, the mutant Mut2D group exhibited a faster reaction rate and yielded higher DAG content compared with the wild-type ATL group, suggesting its potential for further development as an immobilized biocatalyst for functional lipid synthesis in the industrial process. Furthermore, this study might provide valuable references for the molecular engineering of other lipase superfamily members.

## Figures and Tables

**Table 1:** Thermostability of wild-type ATL and disulfide bond mutants.

Enzyme	*T_m_*	Δ*T_m_*	*t*_1/2_ (min) ^1^	*T_opt_* (°C)
Wild-type	50.68 ± 0.05		28.7 ± 2.3	45
A46C-N68C	54.77 ± 0.19 *	4.09	311.5 ± 21.7 *	50
A56C-S82C	51.24 ± 0.11 *	0.56		
V66C-A297C	51.43 ± 0.15 *	0.75		
S84C-A92C	52.19 ± 0.04 *	1.51	130.2 ± 15.9 *	45
T89C-A143C	50.48 ± 0.08	−0.20		
A249C-Q259C	52.99 ± 0.04 *	2.31	242.6 ± 18.7 *	45
D251C-W290C	46.53 ± 0.06 *	−4.15		
L263C-G271C	52.84 ± 0.04 *	2.16	197.3 ± 12.8 *	45
G265C-N268C	47.00 ± 0.13 *	−3.68		
F269C-G274C	47.74 ± 0.09 *	−2.94		
A46C-N68C/L263C-G271C (Mut2D)	56.51 ± 0.07 *	5.83	685.3 ± 39.4 *	50

^1^ Samples were incubated at 45 °C. * indicates *p* < 0.05 compared with the wild-type. Statistical comparisons of the means are performed using one-way ANOVA, and Duncan’s multiple range test is used for multiple comparisons.

**Table 2:** Free thiol contents of wild-type ATL and disulfide bond mutants.

Enzyme	Free Thiol/Molecule (mol/mol)
Wild-type	0.023 ± 0.005
A46C-N68C	0.016 ±0.003
S84C-A92C	0.039 ±0.008
A249C-Q259C	0.028 ± 0.007
L263C-G271C	0.027 ± 0.009
Mut2D	0.033± 0.006

**Table 3:** Kinetic parameters of wild-type ATL and disulfide bond mutants ^1^.

Enzyme	Wild-Type	Mut2D
*K_m_* with pNP-butyrate (μM)	75.3 ± 3.5	68.5 ± 2.6
*k_cat_* with pNP-butyrate (s^−1^)	28.9 ± 1.3	35.4 ± 2.8 *
*K_cat_/K_m_* with pNP-butyrate (s^−1^mM^−1^)	383.7	516.8
*K_m_* with pNP-caprate (μM)	51.7 ± 2.8	47.3 ± 3.7
*k_cat_* with pNP-caprate (s^−1^)	38.4 ± 2.2	44.6 ± 3.5 *
*K_cat_/K_m_* with pNP-caprate (s^−1^mM^−1^)	742.7	943.0
*K_m_* with pNP-stearate (μM)	68.4 ± 1.5	59.7 ± 2.2 *
*k_cat_* with pNP-stearate (s^−1^)	29.1 ± 1.2	35.3 ± 3.1 *
*K_cat_/K_m_* with pNP-stearate (s^−1^mM^−1^)	425.3	591.3

^1^ Kinetic parameters are assessed under conditions of 45 °C and pH 6.0. * indicates *p* < 0.05. Statistical analyses between wild-type and Mut2D are carried out with Student’s *t*-test.

## Data Availability

The data supporting the findings of this study are available from the corresponding author upon reasonable request.

## Supplementary Materials

The following supporting information can be downloaded at: https://www.mdpi.com/article/10.3390/foods15213866/s1, Figure S1: The SDS-PAGE analysis of purified enzymes. M is the molecular mass marker. Lane 1 to 12 are Wild-type, A46C-N68C, A56C-S82C, V66C-A297C, S84C-A92C, T89C-A143C, A249C-Q259C, D251C-W290C, L263C-G271C, G265C-N268C, F269C-G274C and Mut2D, respectively; Figure S2. DAG content comparison of wild-type and Mut2D at their respective optimum temperatures. Statistical comparisons of DAG content between wild-type ATL and mutant Mut2D at their respective optimum temperatures were performed using one-way ANOVA, and Duncan’s multiple range test was used for multiple comparisons. Distinct letters denote significant differences at *p* < 0.05; Figure S3. Average DAG productivity of wild-type and Mut2D. For each time point, the average DAG productivity was calculated using the total reaction time up to that time point. Statistical analyses were carried out with Student’s *t*-test. * indicates *p* < 0.05; Table S1: Primers used in this study.
