# Supplementary material for: Improving the Thermostability of Lipase ATL by Engineering Disulfide Bonds in Flexible Regions
Source: Foods. 2026 Oct 29;15(21):3866. doi: 10.3390/foods15213866 (PMC13763107; doi:10.3390/foods15213866)
Supplement: Supplementary file 1 [file foods-15-03866-s001.zip › foods-4597140-supplementary.pdf]

Supplementary data

Improving the Thermostability of Lipase ATL by Engineering Disulfide Bonds in Flexible Regions

Lilang Li [1,2], Ruiguo Cui [3], Dong Huang [1,2], Naiyong Xiao [1,2] and Fengjun Li [1,2,*]

[1] Guangdong Provincial Key Laboratory of Aquatic Product Processing and Safety, Guangdong Province Engineering Laboratory for Marine Biological Products, Guangdong Provincial Engineering Technology Research Center of Seafood, Guangdong Provincial Engineering Technology Research Center of Prefabricated Seafood Processing and Quality Control, College of Food Science and Technology, Guangdong Ocean University, Zhanjiang 524088, China; langll@gdou.edu.cn (L.L.); h3064866856@163.com (D.H.); nyxiao@gdou.edu.cn (N.X.)

[2] Collaborative Innovation Center of Seafood Deep Processing, Dalian Polytechnic University, Dalian 116034, China

[3] College of Food Science and Technology, Hebei Normal University of Science & Technology, Qinhuangdao 066612, China; ruigc_hnust@163.com

* Correspondence: fengjunli@gdou.edu.cn

Table S1. Primers used in this study.

Primer	Sequence (5′–3′)
A46C-F	TTAGGACAGTAGGTTGCGCAAGCGTATTGAACCCAGAACTTAAAATCTTCA
A46C-R	TGAAGATTTTAAGTTCTGGGTTCAATACGCTTGCGCAACCTACTGTCCTAA
N68C-F	GCTTCCACGTCTGGACAGCAACCTACACTGCAGTTAAG
N68C-R	CTTAACTGCAGTGTAGGTTGCTGTCCAGACGTGGAAGC
A56C-F	AGTTAAGTTTCTCTCCATCTTTGCAAACATAGTTGTTAGGACAGTAGG
A56C-R	CCTACTGTCCTAACAACTATGTTTGCAAAGATGGAGAGAAACTTAACT
S82C-F	TATAGTATCGTCTGAAAAGCATAGCTTGACGGTTGAACC
S82C-R	GGTTCAACCGTCAAGCTATGCTTTTCAGACGATACTATA
V66C-F	CCACGTCTGGACAGTTACCGCAACTGCAGTTAAGTTTCTCTCCAT
V66C-R	ATGGAGAGAAACTTAACTGCAGTTGCGGTAACTGTCCAGACGTGG
A297C-F	CGGGTCCTTTACAACAATCAGCGTGAATGAAGTACCACACG
A297C-R	CGTGTGGTACTTCATTCACGCTGATTGTTGTAAAGGACCCG
S84C-F	GTCCGTTATAGTATCGTCGCAAAAGGATAGCTTGACGGTTGAAC
S84C-R	GTTCAACCGTCAAGCTATCCTTTTGCGACGATACTATAACGGAC
A92C-F	CAGCGACAAAACCACAAGTGTCCGTTATAGTATCGTCTGAAAA
A92C-R	TTTTCAGACGATACTATAACGGACACTTGTGGTTTTGTCGCTG
T89C-F	CAAAACCAGCAGTGTCGCATATAGTATCGTCTGAAAAGGATAGCTTGAC
T89C-R	GTCAAGCTATCCTTTTCAGACGATACTATATGCGACACTGCTGGTTTTG
A143C-F	CTCGAACGACTTTCCAACAAGTCCAGAAGCCCAGCT
A143C-R	AGCTGGGCTTCTGGACTTGTTGGAAAGTCGTTCGAG
A249C-F	GTAGTGTTGTCGGGGCATGTAATATAGTATTCAGGAGAAATATGGA
A249C-R	TCCATATTTCTCCTGAATACTATATTACATGCCCCGACAACACTAC
Q259C-F	ATAGCCGTCCAACACGGTTACGCAGTTGTCAGTAACTGTAGTGTT
Q259C-R	AACACTACAGTTACTGACAACTGCGTAACCGTGTTGGACGGCTAT
D251C-F	GTTGTCAGTAACTGTAGTGTTGCAGGGGGCTGTAATATAGTATTCA
D251C-R	TGAATACTATATTACAGCCCCCTGCAACACTACAGTTACTGACAAC
W290C-F	CATCAGCGTGAATGAAGTAGCACACGTGAGAATGAAAAG
W290C-R	CTTTTCATTCTCACGTGTGCTACTTCATTCACGCTGATG
L263C-F	CTTAAAATTAACATAGCCGTCGCACACGGTTACCTGGTTGTCAG
L263C-R	CTGACAACCAGGTAACCGTGTGCGACGGCTATGTTAATTTTAAG
G271C-F	CGGATGTACCTGTATTGCACTTAAAATTAACATAGCCGTC
G271C-R	GACGGCTATGTTAATTTTAAGTGCAATACAGGTACATCCG
G265C-N268C-F	ACCTGTATTGCCCTTAAAACAAACATAGCAGTCCAACACGGTTACCTGG
G265C-N268C-R	CCAGGTAACCGTGTTGGACTGCTATGTTTGTTTTAAGGGCAATACAGGT
F269C-G274C-F	CGTGTTGGACGGCTATGTTAATtgtAAGGGCAATACAtgtACATCCGGAGGTTTACCTG
F269C-G274C-R	CAGGTAAACCTCCGGATGTacaTGTATTGCCCTTacaATTAACATAGCCGTCCAACACG

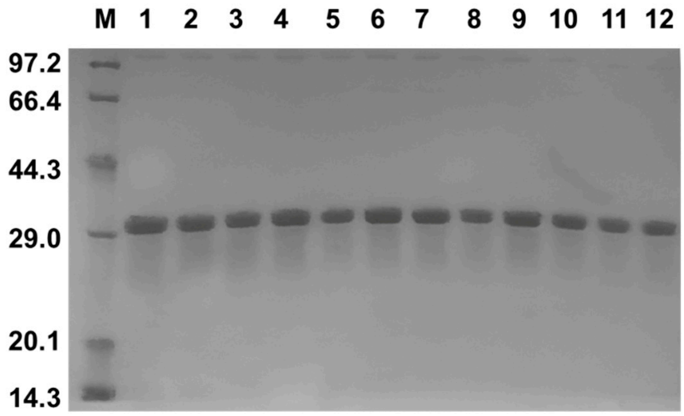

Figure S1. The SDS-PAGE analysis of purified enzymes. M is the molecular mass marker. Lane 1 to 12 are Wild-type, A46C-N68C, A56C-S82C, V66C-A297C, S84C-A92C, T89C-A143C, A249C-Q259C, D251C-W290C, L263C-G271C, G265C-N268C, F269C-G274C and Mut2D, respectively.

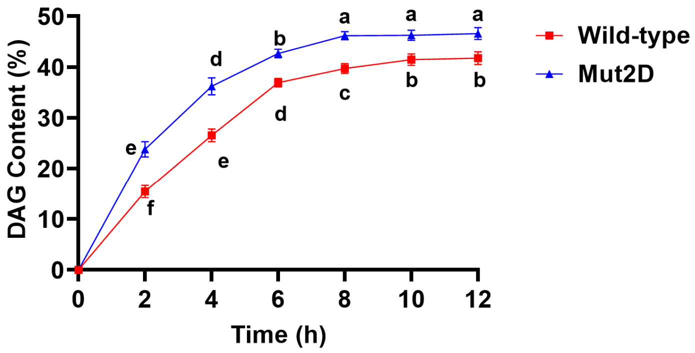

Figure S2. DAG content comparison of wild-type and Mut2D at their respective optimum temperatures. Statistical comparisons in DAG content between wild-type ATL and mutant Mut2D at their respective optimum temperature were performed using one-way ANOVA, and Duncan's multiple range test was used for multiple comparisons. Distinct letters denote significant differences at the $P<0.05$.

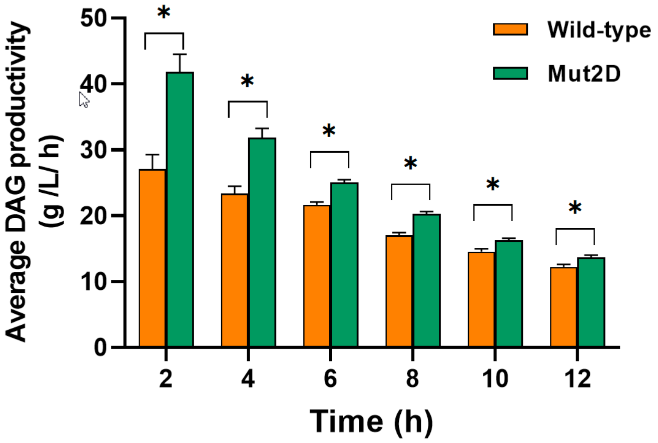

Figure S3. Average DAG productivity of wild-type and Mut2D at their respective optimum temperatures. For each time point, the average DAG productivity was calculated using the total reaction time up to that time point. Statistical analyses were carried out with the Student's t-test. * indicates $P < 0.05$.
